# Cytokine Storm in a Massively Burned Pediatric Patient

**DOI:** 10.1093/jbcr/iraf007

**Published:** 2025-01-17

**Authors:** Mala Sharma, Lauren Roach, Ashleigh Bull, LeeAnne Flygt, Zuhair Ballas, Alex Kurjatko, Madhuradhar Chegondi, Lucy Wibbenmeyer

**Affiliations:** Division of Acute Care Surgery, Department of Surgery, University of Iowa, Iowa City, IA, United States; Division of Critical Care, Department of Pediatrics, University of Iowa Stead Family Children’s Hospital, Iowa City, IA, United States; Division of Acute Care Surgery, Department of Surgery, University of Iowa, Iowa City, IA, United States; Division of Critical Care, Department of Pediatrics, University of Iowa Stead Family Children’s Hospital, Iowa City, IA, United States; Immunology Division, Department of Internal Medicine, Carver College of Medicine, University of Iowa and the Iowa City VA, Iowa City, IA, United States; Division of Acute Care Surgery, Department of Surgery, University of Iowa, Iowa City, IA, United States; Division of Critical Care, Department of Pediatrics, University of Illinois College of Medicine, Peoria, IL, United States; Division of Critical Care, Department of Pediatrics, University of Iowa Stead Family Children’s Hospital, Iowa City, IA, United States; Division of Acute Care Surgery, Department of Surgery, University of Iowa, Iowa City, IA, United States

**Keywords:** burn injury, pediatric burn injury, massive burn injury, cytokine storm, Meek micrografting

## Abstract

Cytokine storm can occur in many different clinical conditions and lack of recognition can lead to death. While cytokines have been measured and trended in burn patients, cytokine storm has not been widely discussed or its treatment reported. We present herein the diagnosis and the treatment of a 5-year-old, 91% burn patient, who developed cytokine storm 3 times during his hospital course. Aggressive treatment led to a successful resolution. Cytokine storm should be entertained in patients who develop classic storm signs and symptoms, along with supporting laboratory. Treatment should be tailored to the patients’ clinical picture. More study is warranted to define cytokine storm as well as its treatment in burn patients.

## INTRODUCTION

Cytokine storm is an ill-defined syndrome characterized by life-threatening, systemic immune dysregulation associated with elevated circulating cytokines.^1^ Cytokine storm is now recognized to be an underlying problem in many hyper-inflammatory diseases including hemolytic hemophagocytosis, macrophage activation syndrome, cytokine release syndrome associated with CAR-T therapy, adult respiratory distress syndrome, immune reconstitution, systemic inflammatory response syndrome (SIRS), and multiple system organ failure (MSOF) among others.^2–4^ Symptoms of cytokine storm can be nonspecific (fever, nausea, vomiting, and diarrhea) or they can be due to hyperinflammation-induced organ damage.^1^ If dysregulated, the persistent inflammatory response can also lead to MSOF and death. Due to the poorly defined nature of this syndrome, and the many physiologic states it can occur in, there is currently no consensus on standard treatment. Depending on the underlying cause, immunosuppressive medications or immunomodulators can be used. However, these medications are not without adverse effects.

While the hyper-inflammatory state of large burn injuries has been well documented in the literature,^5–10^ the actual incidence of cytokine storm and its treatment in burn patients remains unclear. Moreover, there seem to be differences in the burn-induced cytokine profiles in adults and pediatric patients.^7^ Elevations in both the pro- and anti-inflammatory state in pediatric burn injury are commonly seen in the early post-burn period with resolution by the fifth week.^6^ Hyperinflammation in burn patients contributes to hypermetabolism, insulin resistance, and impaired hepatic function with dysregulated triglyceride metabolism (TG).^8,11^ This burn-induced inflammatory syndrome leads to both systemic physiologic and serologic alterations that are typically associated with cytokine storm in other populations complicating the diagnosis in thermally injured patients.^8^

The literature on diagnosing and treating cytokine storms is evolving. We present, to our knowledge, the first case of suspected cytokine storm in a severely injured pediatric burn patient in the absence of a clear underlying cause.

## HOSPITAL COURSE

**Figure 1.**
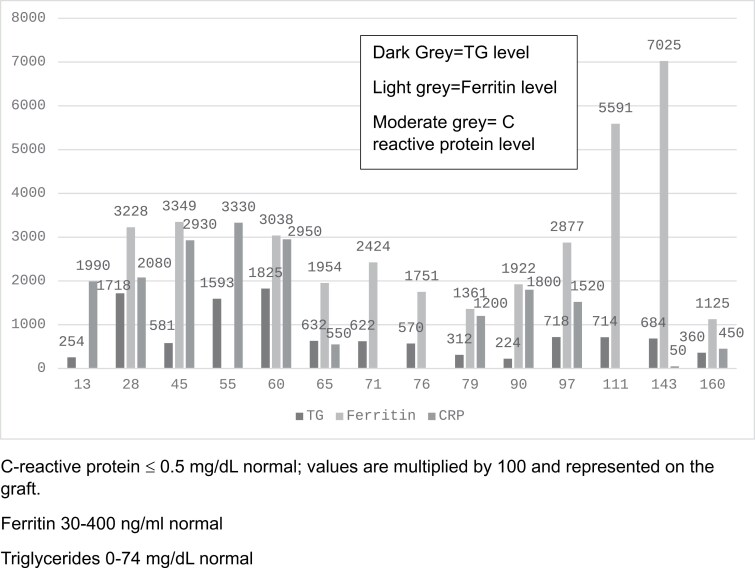
C-Reactive Protein (CRP), Ferritin, and Triglyceride Levels

The patient is a 5-year-old male who presented to the pediatric intensive care unit after sustaining a 91% third-degree flame burn only sparing his belt region, feet, and anterior right leg. Within the first 24 hours of admission, he had a decompressive laparotomy, bilateral canthotomies, and escharotomies to the neck, chest, abdomen, and upper and lower extremities for global decompression. He was resuscitated per Brooke’s formula and received 218 mL/kg or 2.4 mL/kg/percent burn (TBSA) in the first 24 hours. He continued to receive an additional 253 mL/kg or 2.8 mL/kg/TBSA in the next 48 hours. Both vasopressors and stress dose hydrocortisone were provided to support his blood pressure and weaned during the first week of admission. He underwent near complete excision of his burns within 11 days and was placed in polyurethane form. He was taken back to the operating room for several repeat excisions for suspected colonization of his wounds but was not definitively diagnosed or treated for a wound infection. He underwent his first Meek micrografting during his first month and his last one two and one half months after admission. During the first month, he had a central line bloodstream infection with Canada tropicalis. He was also treated for suspected ventilator-associated pneumonia 47 days post admission and received 7 days of antibiotics. No other definitive infection was treated prior to his diagnosis of cytokine storm. Initially, inflammatory markers including ferritin, triglycerides, and c-reactive protein (CRP) were elevated as expected in the hypermetabolic state following the burn injury, however, these markers continued to be elevated for weeks to months following his initial injury. He was also persistently febrile for months following admission. On hospital day 60, he developed a new vasopressor requirement in addition to continued fevers and increasing ferritin, TG, and CRP (see Figure 1). The infectious work-up was negative. He did not have pancreatitis and it had been several weeks since his last propofol administration. Immunology was consulted due to concern for a hyper-inflammatory state. His clinical picture was felt to be consistent with cytokine storm. Cytokine levels showed elevated IL-2, IL-6, IL-8, IL-10, IL-13, and IL-17 (Table 1). The patient was started on intravenous methylprednisolone 1 mg/kg twice a day with treatment escalated to tocilizumab on HD 70 and 97. The patient was also treated with 1 g/kg of intravenous immunoglobulin (IVIG) to keep trough levels above 500 mg/dl. Following this, inflammatory markers initially improved, however, they quickly trended back up with ferritin reaching >6000 almost 4 months after initial presentation. Cytokine panels continued to show elevated but downtrending IL-6 and IL-2 soluble receptors (CD-25). He continued to have persistent fevers as well. Given this constellation of findings, we were unable to wean his steroids and he continued to also receive intermittent treatment with IVIG and tocilizumab (HD 107, 115, 146, and 170).

**Table 1.** Abnormal Cytokine Levels From Hospital Days 60 to 213

Variable^a^	Range (pg/mL)	HD60	HD78	HD108	HD128	HD148	HD168	HD183	HD199	HD 213
IL-10	≤2.8	**15.6**	**13.1**	**15.5**	**9.0**	**4.8**	**32.1**	**11.9**	**12.7**	**31.7**
IL-6	≤2.0	**44.3**	**566**	**46**	**6.7**	**4.9**	**11.2**		**8.7**	**13.8**
IL-13	≤2.3	**2.6**	**2.8**	<1.7	<1.7	<1.7	<1.7	<1.7	<1.7	<1.7
IL-17	≤1.4	**2.1**	<1.4	**1.5**	<1.4	<1.4	<1.4	<1.4	<1.4	<1.4
IL-2r (CD-25s)	175.3-852.8	**2841**	824	**1068**	**1192**	**<858**	**<858**	**1807**	**1456**	**887**
Il-8	≤3	**27.3**	**24.4**	<3	<3	<3	<3	<3	<3	<3

^a^TNF, IL-1b, IL-2, IL-4, IL-5, IL-12, and INF-g were all normal. Bold numbers represent abnormal numbers.

Abbreviations: HD = hospital day; IL = interleukin; mL = milliliter; pg = picogram; r = receptor; s = soluble.

The subsequent episodes of cytokine storm occurred 5 and 6 months after his initial presentation with temperatures greater than 40 °C, profound hypotension requiring multiple vasopressors, and elevated inflammatory markers. The second episode, at 6 months ferritin was at its peak of 28 155 ng/mL. It remained elevated above 1000 for the next 2 months and then finally began to downtrend 9 months after the initial presentation. Other inflammatory markers (CRP and TGs) followed a similar pattern of persistent elevation and then ultimate downtrend. His fever curve mirrored these with near daily fevers for the first 5 months of admission, intermittent but significant fevers in the next 4 months, and then an overall improvement in his fever curve after 9 months.

Throughout the cytokine storm treatment, he underwent autografting with the Meek micrografting method and cultured skin for the majority of his burns. He underwent several repeat excisions for suspected colonization of his wounds but was not definitively diagnosed or treated for a wound infection. He underwent his first Meek micrografting during his first month and his last Meek micrografting two- and one- half months after admission. During the time he was receiving the majority of his immunomodulating treatment, he did not have any clinically significant infections.

## DISCUSSION

While cytokine storm and its treatment have been reported in non-burn critical illness, we present, to our knowledge, the first case of suspected cytokine storm and treatment in a severely injured pediatric burn patient.

Cytokine storm remains poorly defined with criteria varying between disease states. Broadly, cytokine storm is described as a life-threatening syndrome characterized by circulating elevated cytokines and other immune modulators.^2–4^ The syndrome is characterized most often with fever and nonspecific signs, nausea, vomiting, diarrhea, headache, and arthralgias.^1^ If untreated cytokine storm can progress to circulatory shock, MSOF, and death. Treatment, however, can have consequences. Immunosuppressives, either steroids or targeted immune therapy, may hinder the resolution of the primary disease process, ie, the burn injury, and increase susceptibility to infections. Moreover, targeted treatment requires knowledge of the main activated cytokines, which is generally not available at the time of diagnosis.

Diagnosing cytokine storm following a severe burn injury is difficult as severe burns induce a profound hyper-inflammatory and hyper-metabolic state that is necessary for recovery.^8,12,13^ The hypermetabolism of burn injury, driven largely by the immune response, is well characterized. Elevations in catecholamines, glucocorticoids, glucagon, insulin, and dopamine reprioritize metabolism inducing lipolysis, glycolysis, glycogenesis, and proteolysis. Constitutive proteins are downregulated in favor of production of acute-phase reactants.

Burn injuries similarly induce an inflammatory response. The cytokine response after severe burn injuries has been reported but not systematically evaluated. In general, cytokine responses tend to be related to both burn size and age, with larger burns and older age having more exaggerated responses.^5–9,12,14^ The response is maximal in the early phase of burn injury and tapers off after 2 weeks.^6,14^ Studies show that the responses between children and adults may differ.^7^ Finnerty and colleagues showed that both the proinflammatory and anti-inflammatory cytokine responses were maximal during the first week after thermal injury in 19 burned children compared to 14 controls. This contrasts with our patient who showed persistent elevations at 60 days. These authors demonstrated significant increases in 15 mediators in 19 children with burns greater than 40% TBSA including interleukin (IL) -1 beta, IL-2, IL-4, IL-5, IL-6, IL-7, IL-8, IL-10, IL-12 p70, IL-13, IL-17, interferon gamma, monocyte chemoattractant protein 1, macrophage inflammatory protein 1 beta, and granulocyte colony-stimulating factor.^6^ Finally, specific immunomodulators have been associated with either SIRS or death in burn patients including: TNF-alpha, IL-1ra, IL-5, IL-6, IL-8, IL-10, GM-CSF, INF-gamma, and MIP-1beta.^5,12,15–17^

Metabolic changes accompanying cytokine storm in other populations have been described as well and are similar to those seen in severe burn patients including elevations in CRP and ferritin.^8,18^ Our patient demonstrated elevations in both of these markers. In addition, our patient demonstrated profound elevations in TG levels. Elevated TG, along with fatty liver, have been reported in severe burns.^19,20^ Abundance of circulating free fatty acids has been postulated as the reason. However, elevated TGs have been associated with mortality in other disease states as well as burn injury and may be a marker of cytokine storm.^20,21^ Cytokines, especially IL-6, have been implicated in dysregulation of hepatic metabolism and hypertriglyceridemia.^22^

In the absence of infection, the diagnosis of cytokine storm was made in our patient secondary to a constellation of clinical findings and elevations in cytokines, CRP, TG, and ferritin. Steroids were started. With persistent elevations of cytokines, especially IL-6, tocilizumab was given. The patient also received IVIG to boost low levels of IVIG throughout this time period. Therapy was eventually weaned off over several months. Throughout the treatment for his storm, the patient suffered intermittent wound colonizations but no other serious infections. He also did not develop any coagulopathies.

In conclusion, cytokine storm in burn patients is difficult to recognize and its treatment is not without consequences. Early diagnosis is important to prevent sequela. As signs and symptoms are nonspecific and difficult to make in a severe burn patient, cytokine storm should be entertained and evaluated for in burn patients who present with signs of organ system dysregulation and have rising TG, ferritin, and CRP, especially in the absence of an obvious infection. The diagnosis and treatment of cytokine storm in critically ill burn patients needs more focused study.
